# Peroxisome proliferator-activated receptors regulate the progression and treatment of gastrointestinal cancers

**DOI:** 10.3389/fphar.2023.1169566

**Published:** 2023-03-21

**Authors:** Min Zhang, Shujie He

**Affiliations:** ^1^ Department of Cardiology, The First Affiliated Hospital of Nanchang University, Nanchang, China; ^2^ Department of Cardiology, Union Hospital, Tongji Medical College, Huazhong University of Science and Technology, Wuhan, China; ^3^ Hubei Key Laboratory of Biological Targeted Therapy, Union Hospital, Tongji Medical College, Huazhong University of Science and Technology, Wuhan, China; ^4^ Hubei Engineering Research Center for Immunological Diagnosis and Therapy of Cardiovascular Diseases, Union Hospital, Tongji Medical College, Huazhong University of Science and Technology, Wuhan, China

**Keywords:** PPARs, esophageal cancer, cancer progression, gastric cancer, colorectal cancer, cancer treatment

## Abstract

Peroxisome proliferator-activated receptors (PPARs) are essential nuclear hormone receptors regulating metabolic processes, and they participate in the initiation and progression processes of tumors. Gastrointestinal (GI) cancer is a prevalent malignancy worldwide that originates from the tissues of the gastrointestinal tract and is characterized by severe symptoms and poor prognosis. Numerous published studies have investigated the critical role of PPARs in esophageal, gastric, and colorectal cancers. Here, we summarize and review the current literature to understand the role of PPARs in the pathogenesis of GI cancers and to provide a systematic reference for the subsequent investigation and development of efficient therapies targeting PPARs and their pathways.

## Introduction

Gastrointestinal (GI) cancer is a common malignancy worldwide that originates from the gastrointestinal tract and has the characteristics of severe symptoms and poor prognosis (Cui et al., 2021). The GI tract is the most important component of the digestive system, with the functions of food digestion and nutrition absorption, as well as an innate immune system for health monitoring (Fenneman et al., 2023). Malignant transition of the normal of GI tract not only affects the efficiency of nutrition absorption but also causes secondary dysfunction in other organs once metastasis occurs. Specifically, GI cancers include cancers originating from the esophagus, stomach, and colorectum, which together account for about 15 percent of all kinds of cancer (Sung et al., 2021). Thus, it is important to clarify the pathogenesis mechanisms in the progression of GI cancers, which could help provide precise targets for developing efficient therapeutics.

Peroxisome proliferator-activated receptors (PPARs) are nuclear hormone receptors activated by fatty acids to regulate the metabolic processes, with three separate isotypes known as PPARα, PPARγ, and PPARβ/δ (Christofides et al., 2021). Although the three subtypes of PPARs can sense nutritional status and regulate the energy metabolism process, their mechanisms vary significantly. PPARα can regulate the expression of essential enzymes and proteins by regulating the transcriptional process of targeted genes, which further participate in the lipid transportation and *β*-oxidation of fatty acids, resulting in fat degradation (Rigano et al., 2017). PPARγ is widely expressed in different tissues and organs, governing the expression of essential genes using two isoforms of PPARγ, with different lengths, to be activated by agonists (Neschen et al., 2007; Bernardo et al., 2009; Chen et al., 2012; Janani and Ranjitha Kumari, 2015). PPARδ, another isotype, is highly expressed in tissues such as the lung, kidney, and heart, and can regulate the expression of target genes involved in cardiovascular diseases, cancers, and gastrointestinal diseases in combination with progression and treatment at the DNA-binding domain (Ratziu et al., 2016; Kadayat et al., 2020; Xiao and Wang, 2022).

The metabolic processes regulated by PPARs are very important to the function of various organs, and the disturbance of PPARs and related pathways could lead to tumorigenesis, especially in the parts of the gastrointestinal system where nutrition is absorbed. In recent years, many researchers have focused on investigating the role of PPARs in GI cancers and on developing efficient targeted therapeutics (Wu et al., 2016; Xiao and Wang, 2022; Fenneman et al., 2023). In 2012, a team from Italy led by Vittorio Colantuoni systematically summarized and reviewed the roles of PPARs in GI cancers (Fucci et al., 2012). Thus, it is necessary to conclude and summarize the recent published works in the field of PPARs and GI cancers to systematically reveal the implications of PPARs for cancer diagnostics and treatment.

## Regulatory role of PPARs in esophageal cancers

Esophageal cancer (EC) is a prevalent malignant tumor occurring in the mucosal epithelium of the esophagus and includes two main kinds: squamous cell carcinoma (ESCC) and adenocarcinoma (Short et al., 2017). The cancer’s recently published statistical data show that its survival rate is still very low, about 20%, although the overall survival rate for all cancers has increased (Siegel et al., 2022). Unhealthy dietary habits including hot up taking, nutrition deficiency, and alcohol consumption are regarded as essential risk factors for ESCC (van den Brandt, 2022). Risk factors for EC mainly include Barrett’s esophagus, obesity, smoking, and gastroesophageal reflux disease (Lordick et al., 2016; Lagergren et al., 2017). Surgery, chemotherapy, and chemoradiotherapy are the three traditional EC therapies. Neoadjuvant chemoradiotherapy or chemotherapy before surgery is recommended for patients with metastases or at more advanced stages, or who are not suitable for surgery. However, most patients suffer from relapse and poor prognosis (Shah et al., 2020). Therefore, it is urgent to develop more novel and efficient therapeutic tactics for esophageal cancer treatment. With the proposed conception of precise medicine, more and more investigations are exploring the regulatory mechanisms of EC progression, especially the regulatory functions and roles of PPARs.

### The roles of PPARs in regulating esophageal cancers

Yue et al. analyzed esophageal cancer microarray data to profile microRNA expression and construct networks of differentially expressed genes and microRNAs using a bioinformatics approach. The subsequent gene enrichment analysis identified *ADCYAP1R1*, in the PPAR signaling pathway, as potentially involved in the prognosis of esophageal cancer (Chen et al., 2022a). A study conducted by Yukio Terashita et al. examined the correlation between the mRNA expression of PPARγ and esophageal cancer prognosis in 55 patients with primary ESCCs and found that PPARγ expression decreased significantly compared with the normal esophageal epithelium; thus, they concluded that the mRNA expression level of PPARγ could be regarded as a prognosis marker for esophageal cancer patients (Terashita et al., 2002).

### Strategies targeting PPARs to treat esophageal cancers

Targeting the complex regulatory mechanisms of PPARs could provide more opportunities for treatment of ECs. A study investigated the expression profiles of PPARγ in ESCCs and used a novel PPARγ agonist, efatutazone, to treat ESCC cells. The investigators found that the expression of PPARγ was inversely related with Ki-67, and its expression was associated independently with good prognosis in ESCC. In addition, application of efatutazone could inhibit the proliferation of ESCC cells *in vitro* and *in vivo* (Sawayama et al., 2014). Furthermore, a team used the PPARγ agonist rosiglitazone (RGZ) to treat esophageal cancer cells and discovered that application of RGZ could suppress cellular proliferation and induce apoptosis, while PPARγ inhibition could partially restore the RGZ-treated effect, suggesting that activation of PPARγ with specific agonists could inhibit cancer cell growth and cancer progression (Wu et al., 2016). In addition to PPARγ, PPARδ could also act as an essential mediator for 83b1 to exert anti-cancer effects on human esophageal cancers by down-regulating cancer-related genes and molecules (Pun et al., 2017).

## Regulatory role of PPARs in gastric cancer progression and treatment

Gastric cancer (GC) is a severe malignancy and the fifth most common cancer, with about a million new patients suffering this disease every year. It is commonly diagnosed in the late stage, with rapid progression; therefore, the mortality rate remains high despite huge progress in diagnostic equipment and therapies, and it remains the third deadliest cancer in the world (Ferlay et al., 2019). During the proliferation of cancer cells and the progression and metastasis of tumors, large amounts of nutrition and energy are required to support tumorigenesis. PPARs serve as metabolic regulatory mediators, generating energy in response to nutrition stimulation, and play critical roles in the progression of GC. Recently, more and more investigations have focused on the regulatory mechanisms of PPARs, broadening the mechanism networks of GC development (Chen et al., 2022b).

### The roles of PPARs in regulating gastric cancers

A study performed by Yu-Ching et al. investigated the role of visceral-to-subcutaneous fat ratio in GC and found expression of the three isotypes of PPARs to be decreased in patients with higher ratios of visceral adipose tissue *versus* subcutaneous adipose tissue, compared to ones with lower ratios. Furthermore, adipocytes co-cultured with cancer cells could accelerate their proliferation by downregulating the expression of PPARs (Lin et al., 2021). Additionally, recent research has revealed PPAR pathways to be inhibited in patients with gastric carcinoma of the T300A/T300A genotype, accompanied by an increased ratio of tumor cell apoptosis and overall patient survival improvement (Ma et al., 2021). One study investigating the role of N-myc downstream regulated gene 1 (NDRG1) expression in GCs revealed that the downregulation of *NDRG1* indicated malignant behaviors and a poor prognosis, and clarified their relationship with the PPAR signaling pathways (Xiao and Zheng, 2020). Moreover, lncRNA HCP5 derived from MSC could promote the oxidation of fatty acids in gastric cancer cells, thus increasing their stemness and chemo-resistance *via* regulating the signaling axis of miR-3619-5p/AMPK/CEBPB mediated by PPARγ coactivator 1A, demonstrating a novel target of HCP5 as an efficient therapy for GCs (Wu et al., 2020). A team investigating adenomas in early gastric cancer of low-grade dysplasia and high-grade dysplasia analyzed their genomic and transcriptomic landscape and revealed the signaling pathway of PPARs to be decreased significantly in early gastric cancer, while the pathways associated with extracellular matrix and focal adhesion were upregulated (Min et al., 2016). Christie Jeon et al. performed an analysis of the SNPs in PPARs with gastric cancer to illuminate the association between each genotype and gastric cancer and found that PPARδ variants were significantly related to gastric cancer, implicating a role for drugs targeting PPARδ in preventing the progression of gastric cancer (Jeon et al., 2013). In gastric mucosal cells, walnut polyphenol extracts (WPEs) induced nuclear translocation of PPARγ and significantly attenuated the gastric pathologies induced by HP (Park et al., 2020). Similarly, in gastric epithelial cells infected with *Helicobacter pylori* (HP), *β*-carotene could prevent cancer cell invasion by inhibiting the expression of MMP-10 mediated by mitogen-activated protein kinase (MAPK), which is due to increased expression of PPARγ-mediated catalase and reduced levels of ROS (Bae et al., 2021).

### Strategies targeting PPARs to treat gastric cancers

With deep research on the regulatory role of PPARs in gastric cancer, more and more investigations are identifying precise therapies for gastric cancer treatment. Fang Guo *et al*. revealed that cellular proliferation and migration in GC could be inhibited by PPARγ mediating the suppression of TERT and ENAH, implying an important regulatory role for PPARγ and providing solid potential targets for GC therapy (Guo et al., 2016). Another study published recently confirmed that the expression of fatty acid-binding protein 4 (FABP4) in GC patients is positively related to poor outcomes. FABP4 could regulate the translocation of PPARγ to promote the subsequent transcription and expression of cell adhesion molecule 3 (CADM3), which could further inhibit GC metastasis. In addition, application of the PPARγ agonist rosiglitazone could directly activate the downstream effect of FABP4, regulating CADM3 to inhibit the metastasis of GC cells *in vivo* and *in vitro* (Chen et al., 2022b). Meanwhile, a research group reported that the upregulation of PPARα in gastric cancers is negatively correlated with prognosis and that treatment with fenofibrate could regulate PPARα pathways to reverse the dysfunction of mitochondria and cellular metabolism (Chen et al., 2020). In immunotherapy for gastric cancer, PPARγ could induce the expression of DOK1 in macrophages and empower the conversion of macrophages to the inflammatory phenotype, thus inhibiting the viability of gastric cancer cells (Li et al., 2019). A group of researchers tried to develop novel therapeutic drugs targeting PPARs. Using screening methods, Keisuke Yamamoto et al. designed a novel class of PPARγ ligand 1, which is more potent in gastric cancer cells, with a dibenzoazepine scaffold (Yamamoto et al., 2018). Masao Ohashi et al. developed hPPARγ-selective agonist 3 and improved its aqueous solubility to facilitate its application to gastric cancer (Ohashi et al., 2013). In conclusion, these published studies reveal the broad application of PPARs for gastric cancer treatment.

## Regulatory roles of PPARs in colorectal cancer progression and treatment

Colorectal cancer (CRC) is a common malignant tumor of the digestive tract and accounts for the second highest mortality among cancers (Sung et al., 2021). Global health burden analysis of cancers has shown the incidence rate of colorectal cancer to be about 10.2% and the mortality rate to be about 9.2%, which make it a significant global burden. The pathogenesis of colorectal cancer is derived from the uncontrolled proliferation of epithelial cells, which could further destroy the mucosa and invade the deeper tissues, thus affecting the integrity of the colorectal structure (Terzić et al., 2010). Currently, surgery, chemotherapy, and radiotherapy are considered the main treatments for colon cancer, and the choice of treatment relies on the stage of cancer and the specific location. Generally, surgery is suitable for cancers at early stages, while chemotherapy and radiotherapy are applicable for later-stage cancers (Peters, 2019). Furthermore, due to the latent symptoms of CRC, it is common for patients to be diagnosed with CRC at a late stage, which is the leading cause of the high mortality of this disease (Shi et al., 2021). Therefore, it is necessary to pave new avenues for the treatment of CRC by investigating its pathogenesis mechanisms.

### Roles of PPARs in regulating colorectal cancers

Systematic analysis of PPARs by bioinformatics has identified the critical roles of PPARs in the progression and prognosis of CRC. Md Misbah et al. investigated the mechanisms of drug resistance and identified biomarkers of CRC in colon cancer cell lines, revealing PPAR signaling to be one of the pathways enriched by different expression genes (Misbah et al., 2022). In addition, Ozlem Kurnaz et al. confirmed that there is increased risk for patients with the PPARγ T161 allele in CRC development (Kurnaz-Gomleksiz et al., 2022). Additionally, bioinformatics methods were employed to identify genes with different expressions in CRC, and PPAR signaling pathways were demonstrated to play an essential role in the occurrence of colon cancer (Zhang et al., 2022). Regarding the metastatic property of CRC, PPAR signaling pathways were shown to be significantly enriched in metastasis mechanisms by integrating analysis (Liu et al., 2021). Recently, single-cell analysis of primary and metastatic colorectal cancer tumors showed the PPAR signaling pathway to be enriched in the regulatory mechanisms of CRC tumorigenesis and metastasis (Wang et al., 2022).

PPAR signaling pathways play critical roles in colorectal cancer progression. The FABP4 and PPARγ axis has been demonstrated to be a novel target for CRC, while PPARδ has been identified as a tumor suppressor in CRC cells (Zhou et al., 2014; Zhao et al., 2019). More studies have revealed that PPARγ can function as a tumor suppressor in CRC cells *via* regulating miR-145 (Panza et al., 2014) and that PPARγ could induce the expression of PD-L1 in MSS^+^ CRC cells, for which immunogenicity can be enhanced by the pharmacological application of PPARγ agonists (Gutting et al., 2021). Teresa Friedrich et al. studied the role of docking protein 1 (DOK1) in CRC and found PPARγ to be one of the subsequent effectors to mediate the inhibitory effect of proliferation (Friedrich et al., 2016).

### Strategies targeting PPARs to treat colorectal cancers

Apart from the pathogenic role of PPARs, numerous therapies aiming at PPAR signaling pathways have emerged recently. A team systematically analyzed different genes from different colorectal cancer cell lines and identified PPAR as an important indicator affecting radiotherapy sensitivity (Chen et al., 2017). Alessandra Ammazzalorso et al. synthesized novel benzothiazole amides 2b to stimulate the activity of PPARα in order to inhibit the proliferation of CRC cells (2019). N-Acylethanolamine acid amidase (NAAA) was upregulated in CRC patients, and inhibition of NAAA could reduce the proliferation of CRC cells mediated by PPARα (Romano et al., 2022). Moreover, several traditional Chinese drugs were demonstrated to be efficient in the treatment of colorectal cancer. Luteolin could be used as an agonist to activate PPARγ, which can upregulate organic cation/carnitine transporter 2 (OCTN2) to enhance the sensitivity of radiology (Qu et al., 2014); buffalo milk whey was also revealed to have an anti-tumor role through activating the necroptotic pathway and apoptotic pathways, accompanied by the upregulation of PPARγ and the downregulation of PPARα (Cacciola et al., 2022). In conclusion, these published studies systematically revealed the regulatory roles of PPARs in colorectal cancer progression and treatment.

## Conclusion

Genetic regulation, environmental influence, and dietary habits comprehensively contribute to the pathogenesis of cancers. In gastrointestinal cancers, the key role of dietary habits is becoming increasingly important, not only by affecting the local environment of the gastrointestinal tract but also by influencing the entire nutrition metabolism of the body. PPARs are regarded as a kind of central regulatory factor in metabolism and energy conversion, and they regulate diverse aspects of cancer initiation, progression, and prognosis. Overall, PPARs could play protective roles in the homeostasis and function of the gastrointestinal tract, and agonists used to activate PPARs could theoretically inhibit the progression of GI cancers. Some studies have discovered that PPARs could function as promoters of cancers, possibly due to the difference between cancer cells and the tumor stages. Although numerous studies have focused on the roles and mechanisms of PPARs, more studies are still needed to broaden the signaling network and provide more targets for the precise therapy.
